# *Treponema denticola* Major Outer Sheath Protein Induces Actin Assembly at Free Barbed Ends by a PIP2-Dependent Uncapping Mechanism in Fibroblasts

**DOI:** 10.1371/journal.pone.0023736

**Published:** 2011-08-25

**Authors:** Michelle B. Visser, Adeline Koh, Michael Glogauer, Richard P. Ellen

**Affiliations:** Matrix Dynamics Group, Dental Research Institute, University of Toronto, Toronto, Ontario, Canada; University of California Merced, United States of America

## Abstract

The major outer sheath protein (Msp) of *Treponema denticola* perturbs actin dynamics in fibroblasts by inducing actin reorganization, including subcortical actin filament assembly, leading to defective calcium flux, diminished integrin engagement of collagen, and retarded cell migration. Yet, its mechanisms of action are unknown. We challenged Rat-2 fibroblasts with enriched native Msp. Msp activated the small GTPases Rac1, RhoA and Ras, but not Cdc42, yet only Rac1 localized to areas of actin rearrangement. We used Rac1 dominant negative transfection and chemical inhibition of phosphatidylinositol-3 kinase (PI3K) to show that even though Rac1 activation was PI3K-dependent, neither was required for Msp-induced actin rearrangement. Actin free barbed end formation (FBE) by Msp was also PI3K-independent. Immunoblotting experiments showed that gelsolin and CapZ were released from actin filaments, whereas cofilin remained in an inactive state. Msp induced phosphatidylinositol (4,5)-bisphosphate (PIP2) formation through activation of a phosphoinositide 3-phosphatase and its recruitment to areas of actin assembly at the plasma membrane. Using a PIP2 binding peptide or lipid phosphatase inhibitor, PIP2 was shown to be required for Msp-mediated actin uncapping and FBE formation. Evidently, Msp induces actin assembly in fibroblasts by production and recruitment of PIP2 and release of the capping proteins CapZ and gelsolin from actin barbed ends.

## Introduction

Modulation of the host cytoskeleton and its related signalling pathways is a common infection strategy of many bacterial pathogens. Many species act extracellularly; others produce secreted virulence factors that interact directly with host cell cytoskeletal components or cytoskeletal regulatory proteins upon injection. Some virulence factors modulate signal transduction pathways, such as activating or de-activating small GTPases, molecular switches that control many cellular functions, including actin remodelling (for review see [1], [2]). *Treponema denticola* is a prominent oral spirochete associated with polymicrobial periodontal infections and chronic periodontal disease progression [3], [4], [5], and it is known to perturb the actin cytoskeleton of fibroblasts, epithelial cells, and neutrophils.

The major outer sheath protein (Msp) of *T. denticola* is an immunogenic surface antigen [6] which is also associated with released membrane vesicles [7]. Msp displays structural and functional similarity to cytotoxic membrane porins [8], and it is able to bind extracellular matrix proteins [6], [8], [9]. Serum antibodies of humans with periodontal disease recognize Msp, confirming the interaction of this protein with immunogenic cells in the periodontium [10]. *T. denticola* is highly motile due to its flagella [11]; thus along with its innate proteolytic activity [12], [13], [14], *T. denticola* is able to penetrate the epithelium and interact directly with cells in periodontal connective tissues. Msp perturbs actin dynamics in both fibroblasts and neutrophils, leading to impaired cell migration and chemotaxis [15], [16], [17]. Msp also impairs calcium signalling and collagen binding in fibroblasts, as well as causing cell rounding and shrinkage, most likely due to Msp-induced subcortical actin assembly near the plasma membrane [18], [19]. The impact of bacterial proteins like Msp on actin dynamics would be expected to perturb numerous cellular functions that depend on cytoskeletal physiology.

Cell locomotion during inflammatory responses and wound healing requires coordinated cycles of actin assembly and disassembly at the plasma membrane. In fibroblasts, it is well established that migration requires extension of a leading edge lamellipodium along with the formation of adhesion complexes and the coincident detachment of focal complexes in areas trailing the leading edge [20]. Initiation of cell migration involves the formation of actin free barbed ends (FBE), along with subsequent filament elongation by the addition of actin monomers to push the membrane outward and to sustain the leading edge. Thus there is a dynamic “treadmilling” between the monomeric and filamentous actin pools in the cell [21]. In this study, we concentrated on mechanisms by which Msp could affect actin filament assembly in fibroblasts.

Multiple actin binding and severing proteins regulate the formation of FBEs and subsequent assembly of actin filaments. Normally, FBEs are blocked by a capping protein, such as capZ or gelsolin, to prevent erroneous elongation [22], [23], [24]; removal of these proteins creates available FBEs. Nucleation of new actin filaments and subsequent branching of actin networks often occur by proteins of the Arp2/3 complex. Severing of formed actin filaments by proteins, including gelsolin and cofilin, also regulates actin reorganization [23], [25].

Members of the Rho small GTPase family act as molecular switches to control signal transduction pathways in eukaryotic cells, and they are key regulators of the actin cytoskeleton [20], [26]. Their activity varies by cycling between an inactive GDP-bound and an active GTP-bound state [27]. Plasma membrane phosphoinositides also play a key role in actin cytoskeleton regulation. Membrane phosphatidyl (4,5)-bisphosphate (PIP2) acts as a direct regulator of actin binding and capping proteins [28], [29]. PIP2 is also converted to phosphatidyl (3,4,5)-trisphosphate (PIP3) by the action of phsophoinositol-3 kinases (PI3K). PIP3 subsequently acts as a second messenger that regulates small GTPase signalling cascades [30].

In this study, we first hypothesized that Rac1 played a key role in Msp-induced actin rearrangement in fibroblasts. However, though Rac1 was activated, we found subsequently in further experiments that it was not required for actin rearrangement upon fibroblast exposure to Msp. Instead, we describe a novel mechanism that leads to actin FBE formation in fibroblasts: Msp induces *de novo* actin filament assembly through the local production and recruitment of PIP2 by phosphoinositide phosphatase activity and the removal of capping proteins from actin filaments.

## Results

### Msp activates multiple small GTPases in fibroblasts

**Figure 1:**
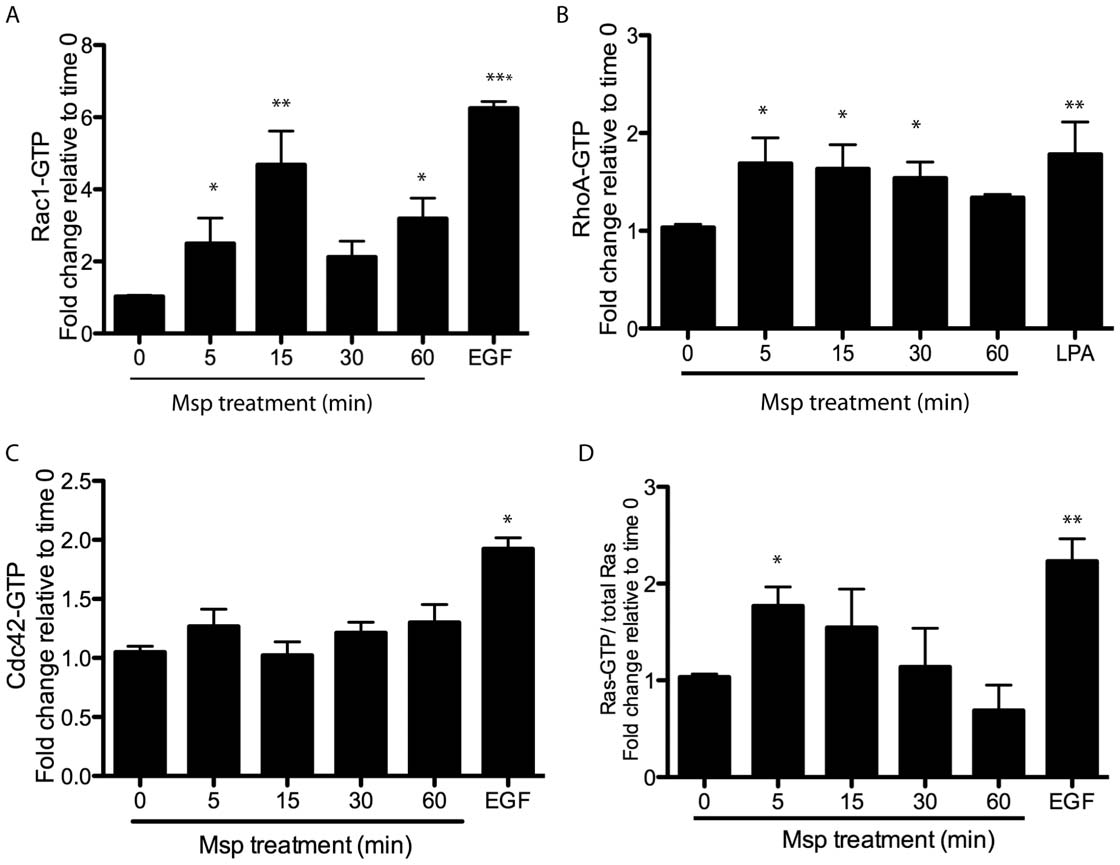
Activation of small GTPases following Msp exposure. Rat-2 fibroblasts were treated with Msp in a time course. At the indicated times, cells were lysed and the level of active Rac1 (A), RhoA (B) or Cdc42 (C) were measured using the appropriate G-LISA. The amount of active Ras (D) in cell lysates was measured using a Raf-RBD pulldown assay. Cells were treated with EGF or LPA as a positive control. Graphs represent the fold change relative to non-treated cells (mean ± SEM, *P<0.05, **P<0.01, ***P<0.001).

We used ELISA-based and pulldown assays to study the impact of Msp on members of the Rho and Ras families of small GTPases. Msp treatment of Rat-2 fibroblasts resulted in activation of Rac1, RhoA and Ras in a time-dependent manner. Each of these small GTPases was activated as early as 5 min following cell exposure to Msp (Figure 1A–C). No significant activation of cdc42 was found (Figure 1D).

### Rac1 localizes in actin-rich areas of Msp-treated fibroblasts

**Figure 2:**
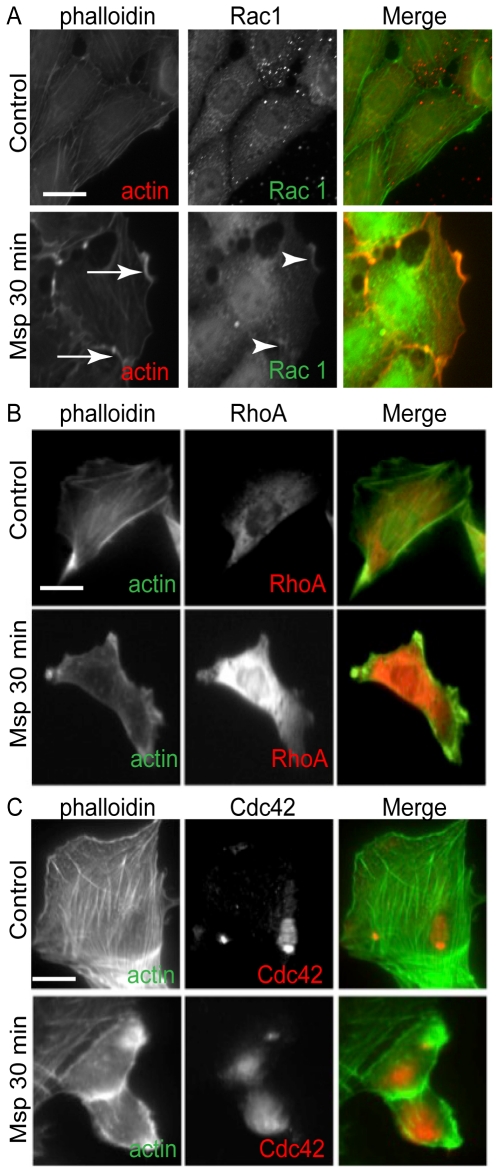
Msp induces re-localization of Rac1 to actin-rich areas. Immunofluorescence microscopy images of fibroblasts following exposure to Msp. Rac1 (A, arrows) is localized to actin-rich areas (A, arrowheads) at the cell periphery. Msp resulted in changed distribution of both RhoA (B) and Cdc42 (C), however neither localized to areas of remodelled actin. Alexa-phalloidin was used to label F-actin while specific antibodies were used to label each small GTPase. Images are representative of 3 experiments. Bar represents 25 µm.

Msp is known to induce *de novo* subcortical actin filament assembly in fibroblasts [15], [18], [19], but its mechanisms of action are relatively unknown. Since the small GTPases, Rac1 and Cdc42 are key regulators of actin remodelling [26] we sought to determine their localization and their relationship to areas of Msp-induced actin reorganization. By immunofluorescence microscopy of phalloidin-labelled cells, we confirmed our laboratory's previous reports that Msp treatment resulted in the loss of stress fibers near the centre of the cell along with the emergence of actin filament-rich areas at the cell periphery. In control cells, Rac1 showed a faint punctate cytoplasmic distribution. Following Msp treatment, Rac1 redistributed to actin-rich areas near the plasma membrane (Figure 2A, arrowheads). In contrast, RhoA and Cdc42 showed more diffuse cytoplasmic distribution following Msp treatment compared with control cells; neither was co-located with actin filaments within actin rich-areas of the cytoplasm (Figure 2B,C).

### Msp-mediated Rac1 activation is PI3K-dependent

**Figure 3:**
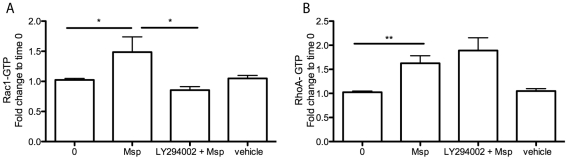
Msp mediated Rac1 activation is PI3K-dependent. Prior to Msp treatment for 15 min, fibroblasts were treated with nothing, vehicle or the PI3K inhibitor LY294002 for 30 min. Activation of Rac1 and RhoA was measured in cell lysates using a G-LISA. Pretreatment with LY294002 was able to inhibit Rac1 activation (A) but not RhoA activation (B) by Msp, compared to Msp treatment alone. Graphs are representative of 3 independent experiments (mean ± SEM, *P<0.05, **P<0.01).

One of the major mechanisms of Rac1 activation involves PIP3 binding to guanine nucleotide exchange factors (GEFs) [30]. As PI3K phosphorylates membrane lipids to produce lipid second messengers, including PIP3, we asked whether inhibition of PI3K would affect Msp-induced small GTPase activation. Pretreatment of fibroblasts with the chemical PI3K inhibitor LY294002 prevented Rac1 activation by subsequent Msp treatment (Figure 3A). This finding suggests that Msp increases PI3K activity upstream of Rac1 activation.

Even though we found that Msp increased RhoA-GTP in fibroblasts, RhoA activation evidently does not occur via the PI3K pathway, as cells pretreated with LY294002 prior to Msp stimulation showed RhoA-GTP activity similar to that of non-LY294002-treated control cells (Figure 3B). Fibroblasts treated with LY294002 prior to subsequent platelet-derived growth factor (PDGF; positive control) stimulation, showed significantly decreased levels of the downstream PI3K effector Akt, confirming inhibition of the PI3K pathway by this chemical inhibitor (data not shown).

### Msp does not activate the PI3K/ Akt signalling pathway

**Figure 4:**
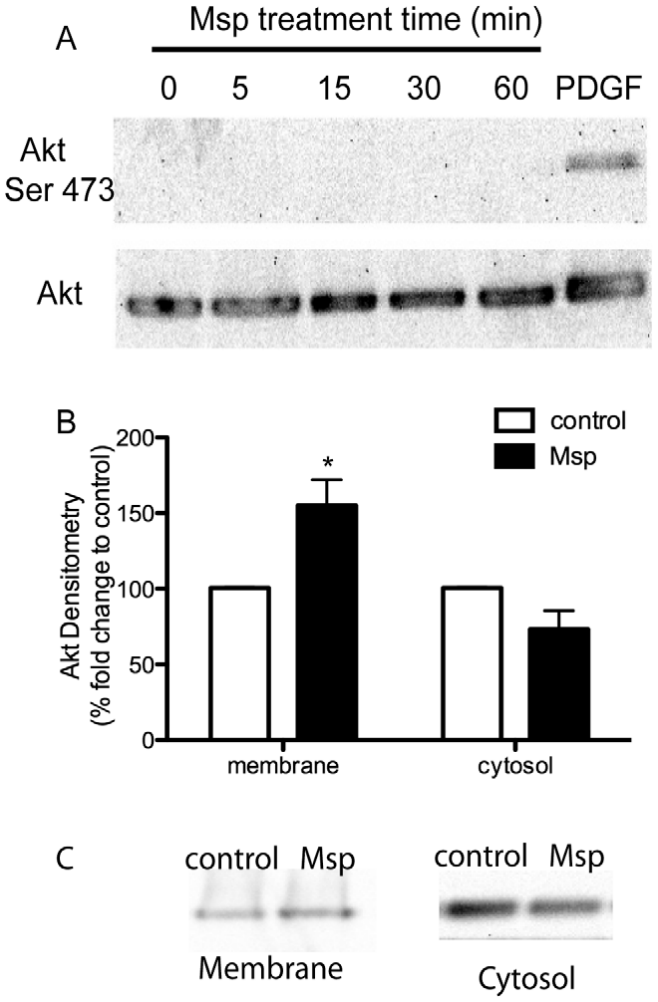
Msp results in Akt translocation but not activation. (A) Msp treated fibroblasts were analyzed for Akt phosphorylation at serine 473 as a measure of activation. No activation of Akt following Msp exposure was observed by immunoblotting. (B) Densitometry analysis of Akt localization to membrane or cytosol fractions following Msp treatment. Graph represents two immunoblotting experiments (mean ± SEM, * P<0.05). (C) Representative immunoblots demonstrating recruitment of Akt to the membrane following Msp treatment. PDGF treatment of cells serves as a positive control for Akt phosphorylation in panel A.

The downstream PI3K effector Akt, may function in actin remodelling [31]. As we had evidence that Msp may activate PI3K, we determined whether Msp acted through Akt to induce actin remodelling. Immunoblotting analysis of cell lysates from a time course of Msp treatment of fibroblasts showed no activation of Akt, measured by phosphorylation at serine 473. Control fibroblasts showed no detectable level of Akt phosphorylation while cells stimulated with 50 µg ml^−1^ PDGF showed strong activation (Figure 4A). Additionally, comparative immunoblotting of fibroblast cytosol and membrane fractions revealed increased association of Akt with the membrane following Msp treatment (Figure 4B,C). Together these results suggest that Msp may result in translocation of Akt to the plasma membrane, but not subsequent Akt activation. Apparently, Msp does not activate the PI3K / Akt arm of the signalling pathway.

### Rac1 is not required for Msp-mediated actin rearrangement

**Figure 5:**
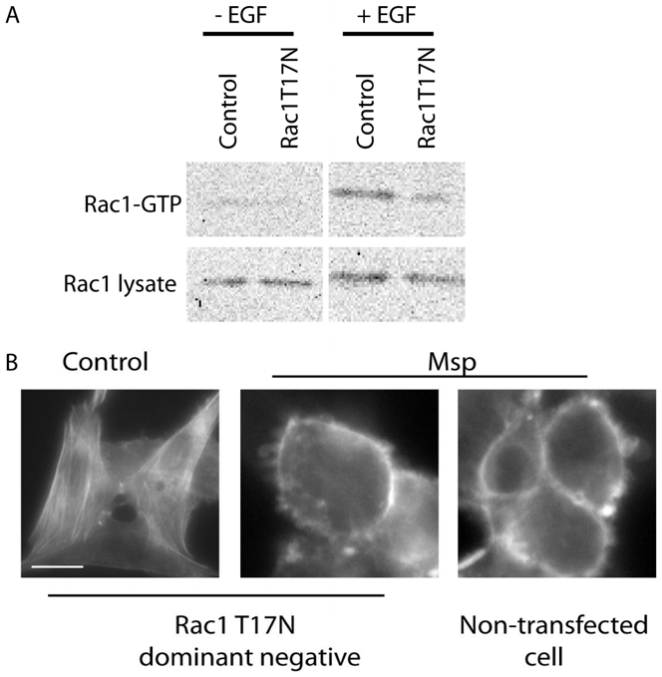
Rac1 is not required for Msp-mediated actin rearrangement. (A) Immunoblot confirmation of Rac1 inhibition following Rac1T17N-GFP transfection. Active Rac1 was measured by pulldown assay following EGF stimulation. (B) Fibroblasts were transiently transfected with a Rac1T17N-GFP construct for 24 h, followed by Msp exposure for 60 min. Samples were fixed and stained with Alexa-phalloidin to label total F-actin. Inhibition of Rac1 activity by transfection was not able to impair subcortical actin remodelling by Msp. Bar represents 10 µm.

As Rac1, a known primary regulator of actin reorganization, was located at the actin-rich areas of Msp-treated fibroblasts, we sought to determine the role of this small GTPase in actin remodelling. To inhibit Rac1 activity, we transfected cells with a Rac1T17N dominant-negative (DN) construct. Rac1 inhibition in transfected cells was confirmed by stimulation with EGF (Figure 5A). Following Msp treatment of transfected cells, actin-rich ruffles and a strong ring of subcortical actin were still observed by Alexa-phalloidin fluorescence, similar to non-transfected cells (Figure 5B). Evidently, Rac1 is not required for actin rearrangement following cell exposure to Msp.

### PI3K is not required for Msp-mediated FBE formation or total actin rearrangement

**Figure 6:**
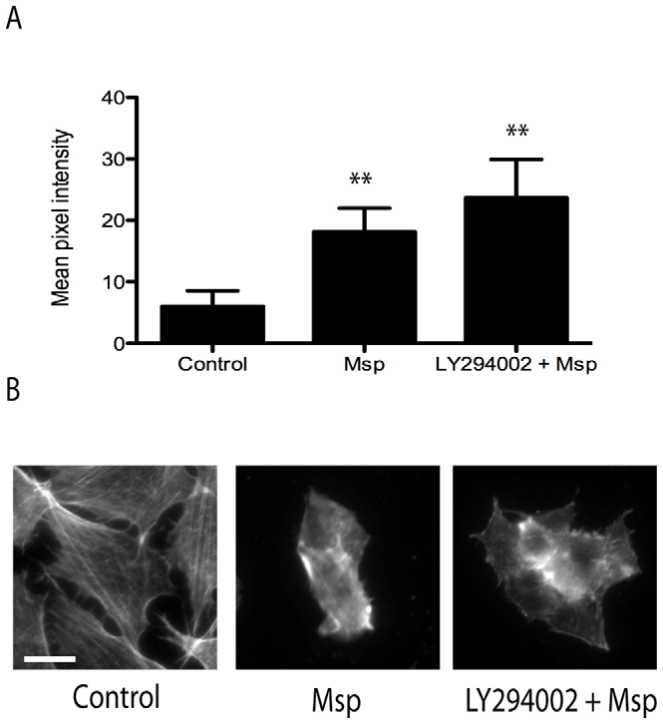
Msp-mediated actin rearrangement and free barbed end formation does not require PI3K activity. (A) Fibroblasts were treated with or without the PI3K inhibitor LY294002 prior to Msp exposure. Cells were partially permeabilized, followed by the addition of rhodamine labelled actin monomers. Incorporation of labelled actin monomers was determined by fluorescence intensity measurement using Image J. Graph represents the mean intensity ± SEM of 3 experiments (compared to control cells, **P<0.01). (B) Representative Alexa-phalloidin stained images of total F-actin with and without LY294002 treatment prior to Msp exposure. Bar represents 25 µm.

Fibroblast treatment with Msp was shown previously to result in increased formation of subcortical actin FBEs [15]. Although Rac1 was apparently not required for Msp-mediated actin rearrangement in the present study, PI3K pathways are also known to regulate actin formation. We used a fluorescent-labelled actin monomer incorporation assay and observation of total F-actin labelling to determine the effect of PI3K inhibition. Treatment of cells with the PI3K inhibitor LY294002 prior to Msp resulted in levels of FBE formation and monomer incorporation similar to Msp treatment alone (Figure 6A). As well, when total F-actin labelling using Alexa-phalloidin was examined; cells treated with Msp alone or with LY294002 and Msp both showed comparably strong subcortical actin fluorescence and cell rounding (Figure 6B). Together, these results indicate that both actin FBE formation and F-actin rearrangement are PI3K-independent.

### Actin rearrangement is partially restored in a *T. denticola* Msp mutant

**Figure 7:**
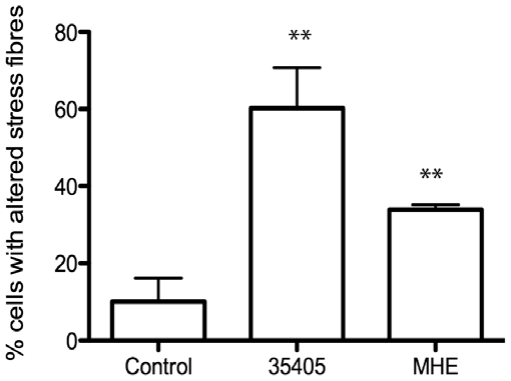
A *Treponema denticola* Msp mutant partially prevents stress fiber alteration. Rat-2 cells were treated with wildtype *T. denticola* 35405 or the Msp mutant MHE for 30 min, followed by F-actin labelling with Alexa-phalloidin. An average of 80 cells per group were counted and the number of cells with altered stress fibres were reported. Graph represents 3 independent experiments (mean ± SEM, ** P<0.01).

To establish the role of Msp in whole bacteria on the fibroblast actin cytoskeleton, we tested wildtype *T. denticola* 35405 and the Msp mutant MHE in an immunofluorescence stress fiber assay. Treatment of fibroblasts with wildtype *T. denticola* increased the number of cells with altered stress fibers, similar to previous studies [32], [33], while the Msp mutant, MHE only partially altered fibroblast stress fibers (Figure 7). This finding indicates that like enriched Msp preparations, Msp in its native bacterial background impacts the host cell actin cytoskeleton, as a Msp mutant strain showed decreased stress fiber disassembly.

### Msp-mediated FBE formation is due to actin uncapping

**Figure 8:**
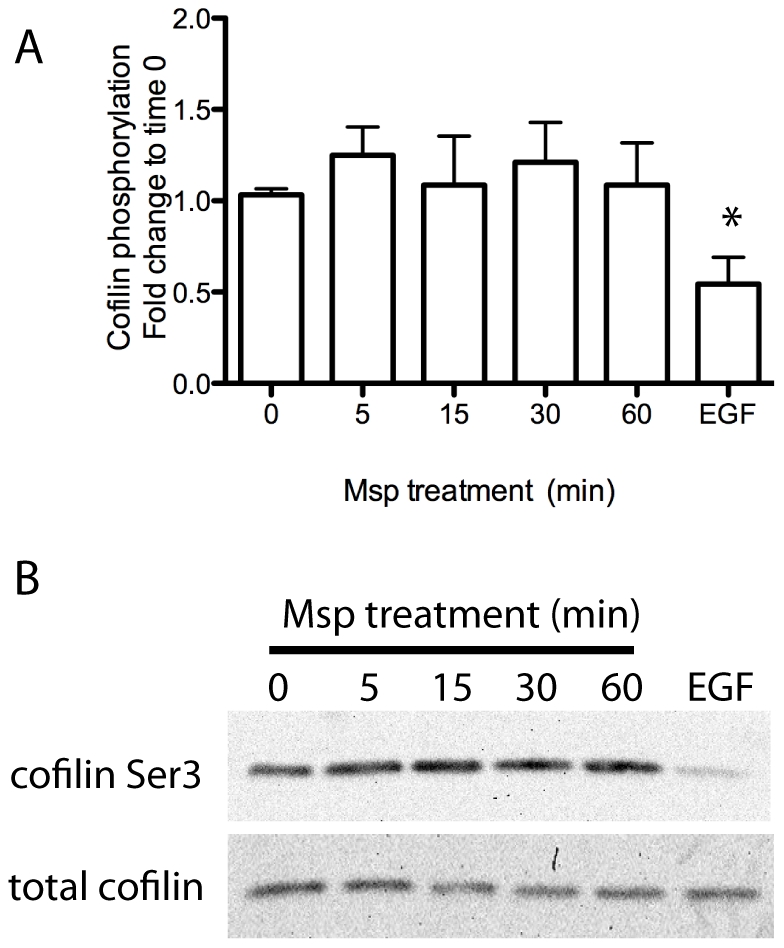
Msp increases cofilin phosphorylation. Cofilin activity as measured by phosphorylation at serine 3 in Msp-treated cell lysates was analyzed by immunoblotting. Densitometry analysis is shown in panel A. Graph represents mean ± SEM of 3 experiments. (*P<0.05). A representative immunoblot is shown in panel B. EGF treated cell lysates serves as a positive control for cofilin dephosphorylation.

The mechanism by which Msp induces actin FBE formation is unknown. FBE formation is regulated by interactions of multiple actin binding proteins. The actin severing protein cofilin is able to cleave pre-existing actin filaments, and thereby generate FBEs when present in the active dephosphorylated form. However, cofilin activity is blocked when phosphorylated at serine 3 [34]. Following Msp treatment, no dephosphorylation of cofilin was observed in cell lysates, as compared with control epidermal growth factor (EGF)-treated fibroblasts, which resulted in obvious activation of cofilin (Figure 8).

**Figure 9:**
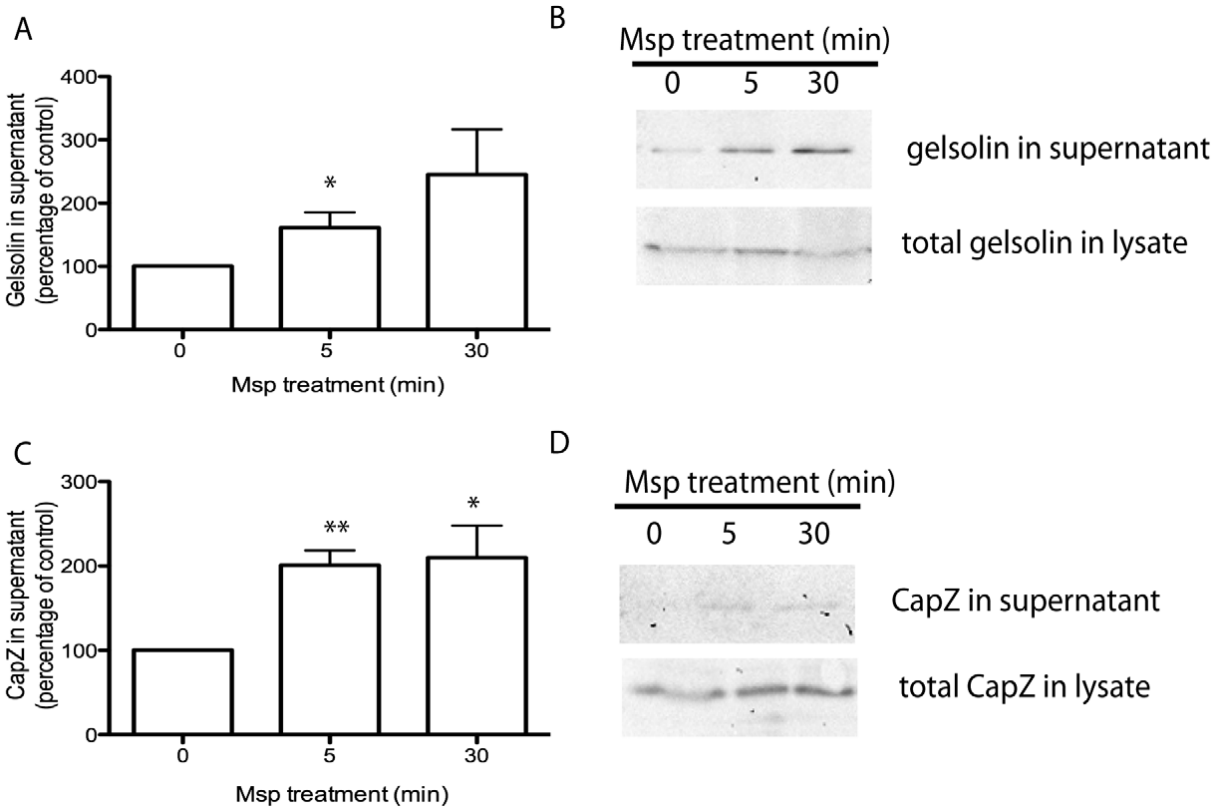
Msp results in uncapping of actin free barbed ends. Fibroblasts were treated with Msp for the indicated times followed by partial permeabilization. The amount of CapZ and gelsolin released from the cell was quantified using immunoblot. Densitometry analysis of released protein is shown (mean ± SEM of 3 independent experiments, *P<0.05, **P<0.01 compared to no treatment) for gelsolin (A) and CapZ (C). Representative immunoblots are shown in panels B and D.

The actin-binding protein gelsolin is able to sever existing filaments as well as cap the newly formed FBEs [24] whereas capping protein CapZ caps the actin barbed end directly [22]. We used immunoblotting analysis to quantify the amount of gelsolin and CapZ released from partially permeabilized cells following Msp treatment. Cell exposure to Msp increased the release of both gelsolin and CapZ from partially permeabilized fibroblasts over time, compared with non-treated control cells (Figure 9). These results suggest that FBE formation induced by Msp is due to the increased removal of actin capping proteins gelsolin and CapZ. We then sought a mechanism to account for this activity.

### Msp induces PIP2 recruitment to actin-rich domains at the plasma membrane and increases PIP2 levels

**Figure 10:**
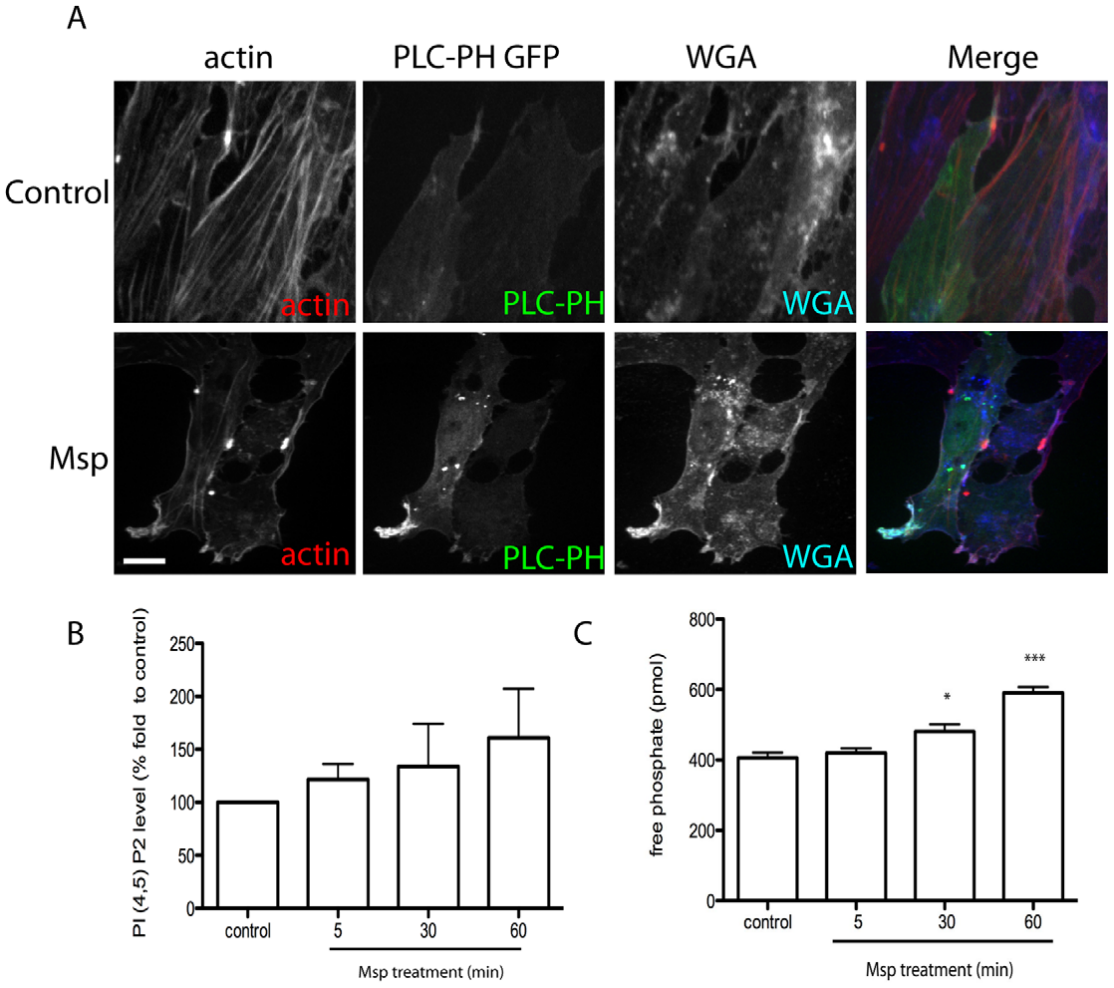
Msp induces PIP2 formation and recruitment to areas of actin remodelling. (A) Rat-2 fibroblasts were transfected with PLCδ1-PH-GFP prior to Msp treatment, fixation and labelling of total F-actin with Alexa-phalloidin and plasma membrane labelling with Alexa-WGA. Msp exposure increased local recruitment of PLCδ1-PH-GFP to actin-rich areas at the plasma membrane. Scale bar equals 25 µm. (B) Total lipids were extracted following Msp treatment and the amount of cellular PIP2 was measured using an ELISA assay. Graph represents the mean ± SEM of 4 experiments. (C) Phosphatase activity was measured using a malachite green assay. Soluble PIP3 substrate was added to supernatant samples from partially permeabilized cells following Msp exposure. The amount of free phosphate released increased following Msp exposure. Graph represents the mean ± SEM of 3 experiments. (*P<0.05, **P<0.01, ***P<0.001).

PIP2 is a well-characterized regulator of the actin cytoskeleton that is able to interact directly with actin binding proteins to regulate their function, including capping of FBEs [29]. To study the dynamics of PIP2 following Msp treatment, we used the pleckstrin homology (PH) domain of Phospholipase C (PLC) δ1 fused to GFP (PH-PLCδ1-GFP) as a fluorescent probe [35]. Following exposure of fibroblasts to Msp, areas of focal PLCδ1-GFP fluorescence were observed, due to recruitment of PIP2, compared to diffuse cytoplasmic localization in control cells (Figure 10A). Co-localization analysis revealed PLCδ1-GFP in areas rich in F-actin and the plasma membrane marker wheat germ agglutinin (WGA) (Fig. 10A, Pearson coefficient analysis (control vs Msp): PLC/actin 0.463 ± 0.037 vs 0.626 ± 0.0261, PLC/ WGA 0.355 ± 0.0405 vs 0.625 ± 0.027). As well, in cells that had become round following Msp exposure, strong fluorescence was also observed along the plasma membrane, similar to localization of the subcortical meshwork of actin filaments (data not shown). To confirm our imaging results we used a PI (4,5) P2 mass ELISA to measure PIP2 levels following Msp treatment. Lipid extracts from Msp treated cells contained increased levels of PI (4,5) P2 compared to extracts from control cells (Figure 10B). Together with these results we analyzed the localization of the PH domain of Akt fused to GFP (PH-Akt-GFP), which is able to bind to PI (3,4,5) P3 [35]. Following Msp treatment, no significant increase in fluorescence for this probe at the plasma membrane was detected, unlike in cells following PDGF stimulation (Figure S1).

Changes in PIP2 levels in the cell can result either from the activity of phosphatidylinositol kinases (PIPK) or phosphoinositide phosphatases [36]. We used a modified malachite green assay in combination with soluble PIP3 as a substrate to measure phosphatase activity. Following Msp treatment, the amount of free phosphate released from partially permeabilized cells increased in a time-dependent manner (Figure 10C).

### PIP2 is required for actin uncapping and actin FBE formation

**Figure 11:**
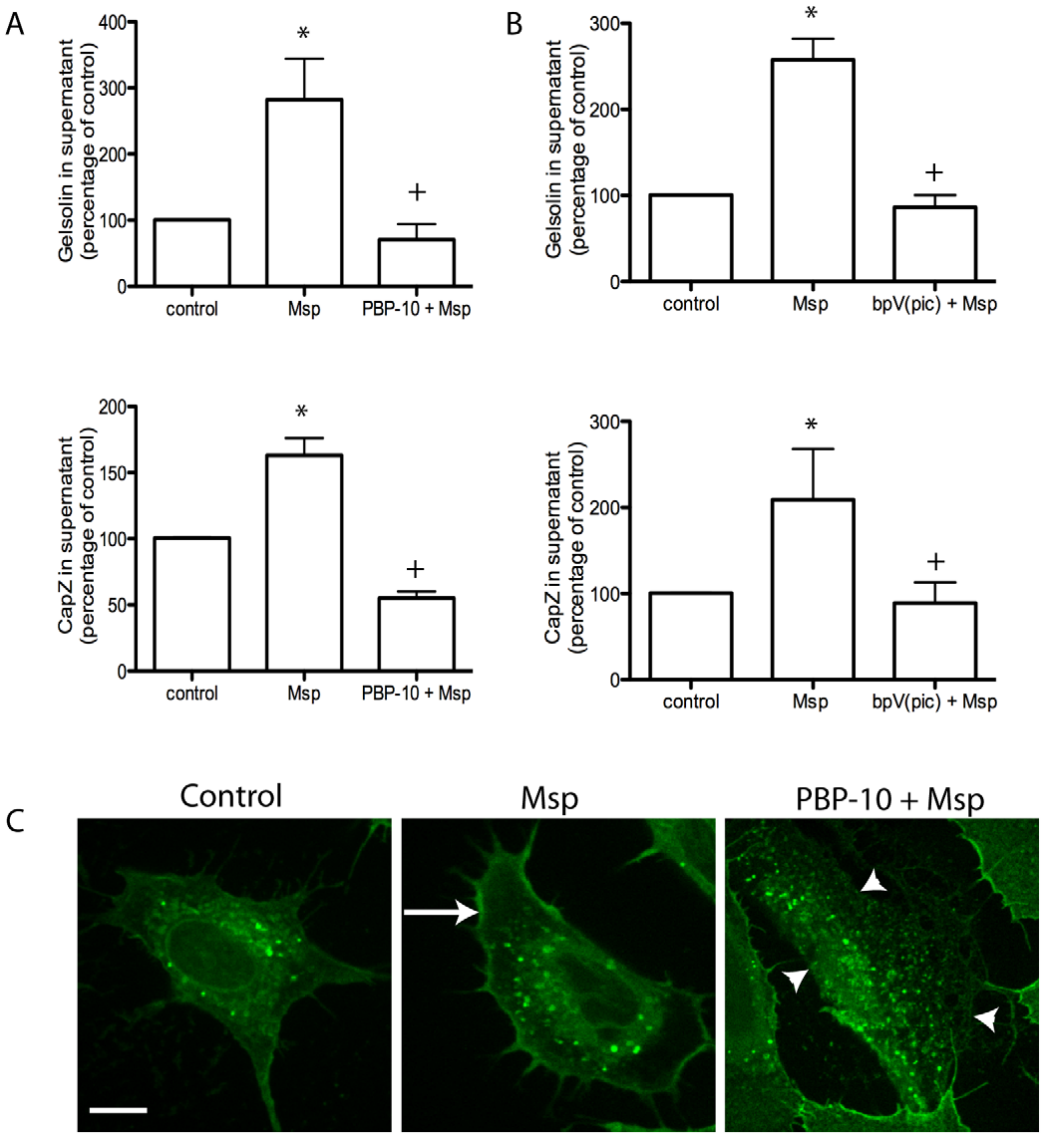
PIP2 is required for Msp-mediated actin uncapping and FBE formation. Pretreatment of cells with the cell permeable phosphoinositide binding peptide PBP-10 (A) or the PTEN phosphatase inhibitor bpV(pic) (B) prevented Msp-mediated release of both gelsolin and CapZ from permeabilized cells. Graphs represent densitometry analysis of 2 experiments (mean ± SEM, * P<0.05 compared to control cells, + P<0.05 compared to Msp treatment). (C) Pretreatment of cells with PBP-10 prevented incorporation of biotin-labelled actin monomers at actin FBE. Representative images are shown.

We used two approaches to confirm that PIP2 is linked to actin uncapping following Msp treatment. Cells were pretreated with the cell-permeable phosphoinositide-binding peptide PBP-10 or the lipid phosphatase inhibitor bpV(pic), prior to Msp. Treatment of fibroblasts with either of these compounds prevented Msp-mediated uncapping, confirming the involvement of PIP2 in this uncapping mechanism (Figure 11A,B, S2).

As this initial experiment showed that PIP2 is required for removal of gelsolin and CapZ from actin ends, we also used this approach to confirm a link between the uncapping proteins and actin FBE formation. Cells treated with Msp showed strong fluorescence along the cortical edge (Figure 11C, arrow) while cells treated with PBP-10 prior to Msp treatment showed no fluorescence at the cortical edge (Figure 11C, arrowheads). This finding indicates indirectly that gelsolin and CapZ are required for actin FBE formation. In the absence of PIP2, removal of these proteins from actin ends does not occur and consequently FBEs are not formed.

## Discussion

Pathogenic bacteria are able to perturb the eukaryotic cell cytoskeleton through signal transduction systems that initiate upon molecular contact at the plasma membrane or through direct interactions of either shed or secreted proteins that gain entry to the cytoplasm. Previously, our laboratory reported that whole bacteria, outer membrane preparations, as well as the native Msp from *T. denticola* were able to induce actin reorganization in fibroblasts, including a loss of stress fibers [15], [19], [33]. Msp also induced the formation of new actin FBEs [15]. The resultant *de novo* subcortical actin filament assembly was a crucial determinant for two entirely novel downstream effects: 1) conformational inhibition of store-operated calcium influx and 2) inside-out modulation of the affinity of extracellular ligand engagement [18], [19]. In the current investigation, we traced in detail significant pathways and mechanisms that may account for actin filament assembly following Msp induction of FBEs. Our major findings were that 1) Msp stimulates PIP2 production and its local recruitment in fibroblasts, probably leading to the removal of capping proteins from actin filaments to expose barbed ends, and 2) though Msp activates PI3K upstream of small GTPases, neither Rac1 nor PI3K are required for Msp-induced actin rearrangement.

A previous study by our group found that Msp inhibited Rac1 activation selectively in neutrophils, resulting in impaired polarization and chemotaxis in response to a chemoattractant [16]. Yet, by an identical method that analyzed bound GTP, we found that Msp clearly activated Rac1 in a time-dependent manner in fibroblasts, as early as 5 min following Msp treatment, suggesting a role for Rac1 in early signalling events in these stromal cells. It is possible that the difference in responses between these cell types may derive from the expression of Rac2 as well as Rac1 by neutrophils, whereas fibroblasts are known to express only Rac1 [37].

RhoA activation is normally associated with the assembly of actin stress fibers and focal adhesions [38]. Yet, in our current study, treatment with the native Msp complex resulted in the activation of RhoA along with coincident loss of stress fibers. Under some circumstances, Rho signalling may be uncoupled from stress fiber formation in fibroblasts. Ras-transformed fibroblasts lack actin stress fibers, yet also show high levels of RhoA-GTP [39]. It has been determined that prolonged MAPK signalling may also play a major role in stress fiber uncoupling at the level of impairing the response to downstream Rho-kinase function [39]. This led us to analyze the levels of Ras-GTP expression in Msp-treated fibroblasts. Using affinity pulldown assays, we observed increased activation of Ras initially at 5 min following Msp exposure. As well, prolonged activation of the MAPKs p38, ERK [40] and JNK (M.B. Visser, unpublished), maximal between 15 to 30 min, was observed in Msp-treated fibroblasts, suggesting that these mechanisms may be involved in the coincident Msp-induced RhoA activation and observed loss of stress fibers.

The initial steps in cell migration involve formation of a leading edge that is visible as actin-rich membrane protrusions or ruffles, processes that require Rac1 and PI3K. In the early stages of Msp treatment, Rac1 was recruited to the plasma membrane actin-rich areas, some of which appear to be membrane ruffles. This is similar to the actin filament pattern observed in cells expressing activated Rac1 or by external growth factor stimulation [41]. The major mechanism of small GTPase activation involves binding of PIP3 to GEFs [30]. Using chemical inhibition studies, we established that Msp activates the PI3K pathway, determined by its requirement for activation of the indirect downstream effector, Rac1. However, the well established PI3K downstream mediator Akt was not activated by Msp. Our studies here suggest that Msp was able to activate PI3K, which in turn would phosphorylate membrane lipids to produce PIP3. PIP3 could then bind GEFs to translocate and activate Rac1 at the plasma membrane as well as bind the PH domain of Akt to mediate its translocation to the plasma membrane. However, activation of Akt by phosphorylation was not observed. Thus, Msp is able to act at the plasma membrane to activate the PI3K pathway; however, individual downstream components of this pathway are evidently affected differently. How Msp acts at the plasma membrane of fibroblasts is not currently known. However, it is unlikely that Msp penetrates the plasma membrane [19]; Msp probably acts through activation of cell signalling cascades upon extracellular contact with the plasma membrane. For example, Msp has been demonstrated to form pores in model lipid bilayers and HeLa cell membranes, as well as to depolarize and induce acute calcium ion influxes across plasma membranes [19], [42], [43].

Although Rac1 and PI3K are well established in the regulation of actin dynamics, it appears that neither of these elements alone are required for Msp-induced actin rearrangement. Neither chemical inhibition of PI3K nor transfection with a Rac1DN construct was able to prevent F-actin reorganization by Msp. Even though studies have shown that expression of either active Rac1 or PI3K alone are able to cause actin rearrangement in fibroblasts [31], [41], our results suggest that Msp impacts either multiple pathways or acts via a further upstream regulator of actin dynamics.

Actin filament assembly, including FBE formation, is directly regulated by both cytoskeleton binding effectors and local changes in polyphosphoinositides such as PIP2 [29], [44]. It is dependent upon initial formation of FBEs and subsequent elongation of filaments [45]. Previously, we reported that Msp-induced actin FBE formation in both fibroblasts and neutrophils [15]. Here, we demonstrate for the first time that cell exposure to Msp induces the removal of the capping proteins gelsolin and CapZ from actin barbed ends in fibroblasts. We propose that uncapping is the primary mechanism of FBE formation caused by cell exposure to Msp. PIP2 is known to induce dissociation of both gelsolin and CapZ from actin termini in multiple cell types, including platelets and fibroblasts [46], [47], [48]. Our experiments using the PLCδ1-PH-GFP probe to monitor PIP2 dynamics [49] together with an ELISA based assay indicated that Msp increases cellular PIP2 levels and induces recruitment of PIP2 to local areas of actin rearrangement at the plasma membrane. Using a cell-permeant peptide PBP-10, which mimics the PIP2 binding site on gelsolin [50], we showed that PIP2 is required for Msp-mediated actin uncapping. Notably, inhibition of PIP2 binding using the gelsolin-derived peptide, PBP-10, is also able to prevent removal of CapZ from actin filaments. We propose that removal of capping proteins by PIP2 is a key mechanism involved in Msp-mediated actin FBE formation.

Changes in PIP2 levels can result from synthesis by phosphoinositol phosphate kinases (PIPK) [51] or degradation of PIP3 by phosphoinostide phosphatases (PP) [36]. Our studies here, using soluble PIP3 as a substrate, showed that the amount of free phosphate increased following Msp treatment, confirming the involvement of a PP in Msp-mediated PIP2 production. As PLC recognizes the (4,5) PIP2 form rather than the (3,4) PIP2 form [35], PIP3 is probably dephosphorylated at the 3-OH following cell exposure to Msp. The 3- phosphoinositide phosphatase PTEN (phosphatase and tensin homologue deleted on chromosome 10) is able to dephosphorylate PIP3 to PIP2 [52] and is a compelling candidate that may be involved in PIP2 production by Msp. Similarily, our data using a synthetic bisperoxovanadate compound which is able to inhibit multiple phosphatases, including PTEN [53], indicate that PIP2 is formed by Msp through PTEN.

The actin binding protein cofilin has two functions in the cell, to depolymerize actin filaments, resulting in monomer turnover, and to sever filaments to create FBEs. Cofilin is normally inactive in resting cells, inhibited either by binding to PIP2 or by phosphorylation at serine 3 [54]. Our data indicate that cofilin remains inactive due to phosphorylation, following Msp exposure. Small GTPases, including RhoA are also able to activate kinases responsible for cofilin phosphorylation [54], which may be at play following Msp exposure, supported by our observed Msp-induced activation of RhoA. Furthermore, Msp-mediated PIP2 production may also be involved in concurrent cofilin inactivation. Therefore, normal actin dynamics and subsequent cell movement are probably impaired, as activation of cofilin is known to be among the key regulators for protrusion of the cell's leading edge [55], [56].

**Figure 12:**
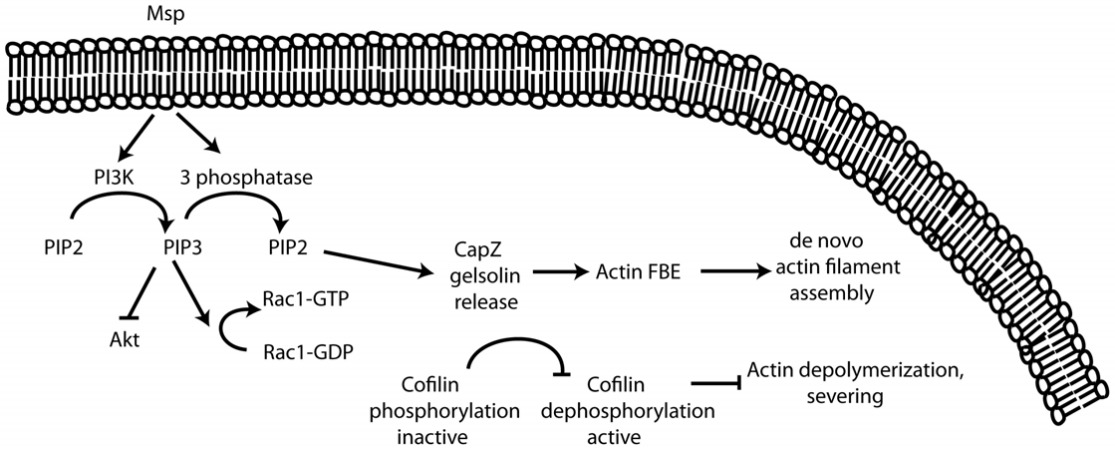
A model for the action of Msp in subcortical actin filament assembly in fibroblasts. Msp treatment of fibroblasts results in activation of both PI3K and a host 3-phosphoinositide phosphatase, resulting in turnover of PIP3 to PIP2. PIP2 then causes removal of actin capping proteins, FBE formation and dependent actin filament assembly, even though cofilin remains inactive. Although Msp activates Rac1, RhoA and Ras, our data suggest that these small GTPases are not directly involved in Msp-induced subcortical actin assembly. 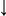 indicates pathway activated, 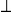 indicates pathway inhibited.

The pathways by which *T. denticola* Msp induces actin filament reorganization appear to be unique among bacterial factors that have been reported to perturb a target host cell's cytoskeleton. Little evidence exists that *T. denticola* invades host cells as a significant feature of its pathogenicity [57]. Similarly, its Msp does not appear to pass through the plasma membrane into the cytosol ([19], M. A. Magalhaes, unpublished). Further we demonstrate here that Msp in its native state in whole bacteria does play a role in host cell cytoskeleton alteration, as a Msp mutant had significantly diminished stress fiber perturbing activity. The analysis of the *T. denticola* 35405 genome has not found any deduced secretion systems which allow penetration of host cell membranes and injection of bacterial proteins into the cytosol (type III, IV or VI), although other general secretion systems to the extracellular environment are present [58]. We suggest that Msp perturbs the fibroblast actin cytoskeleton through a signalling cascade that is novel among those so far reported for bacterial pathogens (Figure 12). Rather than invading or secreting effectors inside the cell to subvert the host machinery, extracellular contact with Msp induces a fibroblast phosphatase that enhances PIP2 production and recruitment, leading to actin filament uncapping and subsequent *de novo* subcortical actin assembly.

## Methods

### Treponema denticola Msp enrichment

Native Msp complex was enriched from broth cultures of *T. denticola* ATCC 35405 as previously described [18], [19] and confirmed by coomassie staining and western blot to be highly pure (data not shown). Msp was used at a concentration of 20 µg ml^−1^ which has been previously determined to promote actin reorganization in fibroblasts [15], [18].

### Fibroblast cell culture

Rat-2 fibroblasts (ATCC CRL 1764) were cultured in α-MEM containing 10% heat- inactivated fetal bovine serum (FBS) and 100 U ml^−1^ penicillinG and 50 µg ml^−1^ gentamicin (Invitrogen). Rat-2 cells from passages 4 through 15 were used for experiments.

### *Treponema* culture conditions and stress fiber assay

*T. denticola* 35405 and the Msp mutant MHE (gift of Christopher Fenno, University of Michigan) were grown as described [59]. For assays, *Treponema* strains were grown for 3 days anaerobically, washed twice with α-MEM, and resuspended to an OD550 of 0.2 (approx. 2×10^9^ cells / ml). Rat-2 cells were grown 18 hr on coverslips in 24-well plates at 9×10^4^ cells / well. One ml of bacterial suspension was added to Rat-2 cells followed by incubation for 30 min 5% CO_2_. Rat-2 cells were washed, fixed and F-actin labelled with Alexa-594 phalloidin. The percentage of cells showing stress fiber reorganization was determined as previously described [15], [33].

### Transfection of fibroblasts

To inhibit Rac1 activity, Rat-2 cells were transfected with a Rac1 dominant negative-enhanced green fluorescent protein (EGFP) fusion (Rac1-T17N). For localization of PIP2 in cells, we used a PH-PLCδ1-GFP fusion construct, while localization of PIP3 was visualized with a PH-AKT-GFP fusion construct (all plasmids were a gift of Sergio Grinstein, SickKids Hospital, Toronto, Ontario). Rat-2 cells were grown in 8-well chamber slides (Lab-Tek, Nunc; 2×10^4^ cells per well) for 24 h, followed by transfection using FuGENE 6 (Roche). The cells were allowed to recover for 24 h prior to treatment with Msp for 60 min in 5% CO_2_ at 37°C. Following treatment, the cells were fixed with 4% paraformaldehye, permeabilized with 0.1% Triton-X100, F-actin labelled using Alexa-594 phalloidin and the plasma membrane labelled with Alexa-647 wheat germ agglutinin (WGA) (Invitrogen).

### Immunofluorescence

To study localization of small GTPases following Msp treatment, 1×10^4^ Rat-2 cells were grown for 48 h in 8 well chamber slides then serum starved for 4 h. Following Msp treatment for 5 or 30 min, cells were fixed with 4% paraformaldehye, permeabilized with 0.1% Triton-X100, and blocked with 5% normal goat serum (NGS) for 30 min at room temperature. Primary antibodies were diluted in 5% NGS and applied for 1 h at room temperature, followed by Alexa-488 goat anti-mouse secondary antibody (Invitrogen). Primary antibodies used were Rac1 (Upstate Biotechnlogy), Cdc42 (BD biosciences) and RhoA (Santa Cruz Biotechnology). F-actin was labelled with Alexa-488 phalloidin and nuclei were stained with 4′,6-diamidino-2-phenylindole (DAPI, Roche).

### Small GTPase activation assays

To determine the activation state of Rac1, RhoA and Cdc42 by Msp, G-LISA assays were used following the manufacturer's instructions (Cytoskeleton Inc). Briefly 2×10^5^ cells were grown in 100-mm dishes for 3 days, followed by serum starvation overnight. Cells were treated with Msp for 5, 15, 30 or 60 min, washed and lysed. Equal amounts of cell lysate samples were used in the G-LISA assays. Rac1 and RhoA activation was measured using a luminescence plate reader (FLUOstar Optima). Cdc42 activation was determined by absorbance values (plate reader model 3550, BioRad).

The Ras activation state following Msp treatment was determined using a Raf-Ras-binding domain (RBD) bound to glutathione agarose beads (Cytoskeleton Inc). 1×10^5^ cells were grown in 150-mm dishes for 4 days, followed by overnight serum starvation. Following Msp treatment as above, cells were lysed and 400 µg of cell lysate was added to 35 µl Raf-RBD beads and incubated for 1 h at 4°C. Bead samples were collected, washed and analyzed by SDS-PAGE. Proteins were transferred to nitrocellulose (Bio-Rad) overnight at 4C. Following transfer, membranes were blocked for 30 min with 5% milk/ TBS/ 0.05% Tween-20, incubated in pan anti-Ras antibody for 3 h at room temperature followed by goat anti-mouse peroxidase-conjugated secondary antibody (Cell signalling Technology). Blots were developed using Western Lighting ECL substrate (Perkin-Elmer) and imaged using a Chemidoc-XRS detector (Bio-Rad). To serve as positive controls, cells were treated for 10 min with 100 ng/ml epidermal growth factor (EGF, Sigma) (Rac1, Cdc42 and Ras) or 10 µM lysophosphatidic acid (LPA, Sigma) (RhoA).

To confirm Rac1 inhibition following Rac1 dominant negative transfection, Rat-2 cells were treated with or without EGF followed by pulldown assay using PAK-GST agarose beads as described previously [16]. Active Rac1 was visualized by immunoblotting as described above using a Rac1 primary antibody (Upstate Biotechnology).

### PI3K inhibition

To study the impact of PI3K inhibition, cells grown in 100-mm dishes were treated with 100 µM LY294002 (Cell Signalling Technology) or DMSO as vehicle control for 30 min at 37°C in 5% CO_2_ prior to treatment with Msp or α-MEM for 15 min. Following Msp treatment, lysates were prepared and Rac1 and RhoA activation levels were measured using the appropriate G-LISA as above (Cytoskeleton).

### Actin Free Barbed End assay

The generation of actin FBEs was determined by incorporation of rhodamine-labelled actin monomers as previously described, with slight modifications [15]. Rat-2 cells were grown in 8-well chamber slides followed by serum starvation for 4 h. In some experiments, the PI3K inhibitor LY294002 was added for 1 h, the rhodamine labelled peptide, PBP-10 for 10 min or vehicle followed by Msp treatment for 30 min. Cells were simultaneously permeablized and labelled with rhodamine muscle actin monomers (Cyotskeleton Inc) by exposure for 30 s to 100 µl of 0.5 µM rhodamine actin in OG buffer (0.4% n-octyl-β-glucopyranoside in PHEM buffer (60 mM PIPES, 25 mM HEPES, 10 mM EGTA, 2 mM MgCl_2_, 0.1 mM ATP pH 6.9). Permeabilization was stopped by adding 300 µl of buffer B (1 mM Tris, 1 mM EGTA, 2 mM MgCl_2_, 10 mM KCl, 5 mM ATP, 5 mM β-mercaptoethanol pH 7.4) for 2 min at room temperature. Cells were washed, fixed for 10 min and permeabilized with 0.1% triton for 5 min. F-actin was labelled with Alexa-488 phalloidin for 20 min at room temperature. Slides were mounted using Prolong Gold medium (Invitrogen). FBE assays following PBP-10 treatment were performed as above except cells were labelled with biotin muscle actin monomers (Cytoskeleton) and Alexa-633 Streptavidin (Invitrogen) was used to visualize FBEs. Fluorescence intensity was measured using ImageJ.

### Image acquisition

Cells were examined by epifluorescence microscopy (Leica DMIRE2) and images obtained using OpenLab software (Perkin Elmer) or by spinning disk confocal microscopy (Leica DMIRE2 with a spinning disk confocal scan head) and images obtained using Volocity software (Perkin Elmer).

### Actin binding protein quantification

The phosphorylation state of the actin binding protein cofilin was determined following Msp treatment. Cells were grown and treated as described above. Equal amounts of cell lysates were analyzed for phospho-cofilin (Serine 3, Cell Signalling Technology) levels by western blotting followed by HRP inactivation and reprobing with a total cofilin antibody (Cell Signalling Technology). As a positive control, cell lysates were used from Rat-2 cells treated with 100 ng/ml EGF for 5 min.

The amount of gelsolin and CapZ present in the supernatant of partially permeabilized cells and total cell lysate was also determined. Confluent fibroblasts in 100-mm dishes were treated with Msp for 5 or 30 min, washed and permeabilized with 400 µl of 0.4% OG buffer for 30 s. The supernatant was collected and concentrated using amicon devices (10,000 MWCO, Millipore) and separated by SDS-PAGE. The cells were scraped from the plate, lysed by passage through a 21 gauge needle, and resuspended in SDS sample buffer followed by SDS-PAGE. Following transfer to a nitrocellulose membrane, samples were blotted with anti-gelsolin (gift of Christopher McCulloch, University of Toronto) or anti-CapZ antibodies (BD biosciences). In some experiments, cells were treated with 30 µM polyphosphoinsoitide-binding peptide PBP-10 [50] (Calbiochem) for 10 min or 100 nM PTEN inhibitor bpV(pic) [53] (Santa Cruz) for 30 min prior to Msp treatment.

### Akt analysis

Rat-2 fibroblasts were grown in 60-mm, 100-mm dishes or 8-well chamber slides as above and treated with Msp. For western blot analysis, equal amount of total protein lysates were separated by SDS-PAGE, followed by transfer to PVDF. Membranes were blocked in 5% milk/ TBS/ 0.1% Tween-20, incubated overnight in primary antibody (anti-Akt Serine 473, Cell Signalling Technology) followed by secondary antibody. Following development, HRP was inactivated with 0.2% sodium azide and reprobed with an anti-non-phosphorylated Akt antibody (Cell Signalling Technology). Crude membrane and cytosol fractions were prepared as follows. Cells were scraped from the dish with cold cytosol buffer (20 mM Tris, 150 mM NaCl, pH 7.5, protease inhibitor cocktail (Sigma)), followed by 3 cycles of freezing in liquid nitrogen and thawing at 37°C. Samples were centrifuged and the supernatant represented the cytosol fraction. The pellet was washed in cytosol buffer, centrifuged and suspended in membrane buffer (cytosol buffer containing 1% Triton X-100). Following centrifugation, the supernatant represented the membrane fraction. Samples were analyzed by immunoblotting as above.

### Phosphatase activity assay

The amount of free phosphate was determined using a modified malachite green phsophatase assay. 1×10^5^ cells were grown per well in 12-well plates (Corning) for 24 h following by serum starvation overnight. Cells were treated with Msp prepared in phosphate-free HBSS, followed by permeabilization with 0.1 volume of 2% OG for 15 s. Sixty µl of solution was removed, to which 1 µM of Phosphatidylinositol 3,4,5-trisphosphate diC8 (Echelon) was added as a substrate. Twenty five µl of this mixture was incubated with 100 µl of malachite green solution (Echelon) in a 96 well plate for 10 min. Absorbance at 650 nm was measured using a microplate reader (VERSAmax plate reader, Molecular Devices Corporation) and the amount of free phosphate calculated using a prepared phosphate standard curve.

### PIP2 assay

PI(4,5)P2 levels were measured in cell extracts using a competitive binding ELISA (Echelon). 3×10^5^ cells were grown per well in 6-well plates overnight followed by serum starvation. Cells were treated with Msp in a timecourse, followed by lipid extraction according to the manufacturer's protocol. Dried samples were resuspended by sonication and assayed in duplicate.

### Statistical analysis

Comparisons between two groups were performed using t-tests. Multiple sample comparisons were performed using one-way ANOVA, with a Dunnett multiple comparison test. Results are based on at least 3 independent experiments. Statistical significance was defined as *P*<0.05. For colocalization studies, Pearson coefficient analysis of dual-labelled images of the same cells was performed using ImageJ.

## Supporting Information

Figure S1 **Localization of Akt-PH-GFP following Msp treatment.** Rat-2 fibroblasts were transfected with Akt-PH-GFP prior to Msp treatment and fixation. Representative images are shown in panel A. (B) Fluorescence intensity at the plasma membrane was quantified using ImageJ. PDGF treatment serves as a positive control for recruitment to the plasma membrane. Graph represents mean ± SEM of 3 experiments (P<0.05).
(TIF)

Figure S2 **Treatment of fibroblasts with a PIP2 binding peptide or a lipid phosphatase inhibitor prevents Msp- mediated actin uncapping.** Rat-2 cells were pretreated with (A, C) PBP-10 or (B, D) bpV(pic) prior to Msp treatment. Representative immunoblots of the amount of released gelsolin (A, B) and CapZ (C,D) are shown.
(TIF)
