# Supplementary figures and images for: Treponema denticola Major Outer Sheath Protein Induces Actin Assembly at Free Barbed Ends by a PIP2-Dependent Uncapping Mechanism in Fibroblasts
Source: PLoS One. 2011 Aug 25;6(8):e23736. doi: 10.1371/journal.pone.0023736 (PMC3161991; doi:10.1371/journal.pone.0023736)

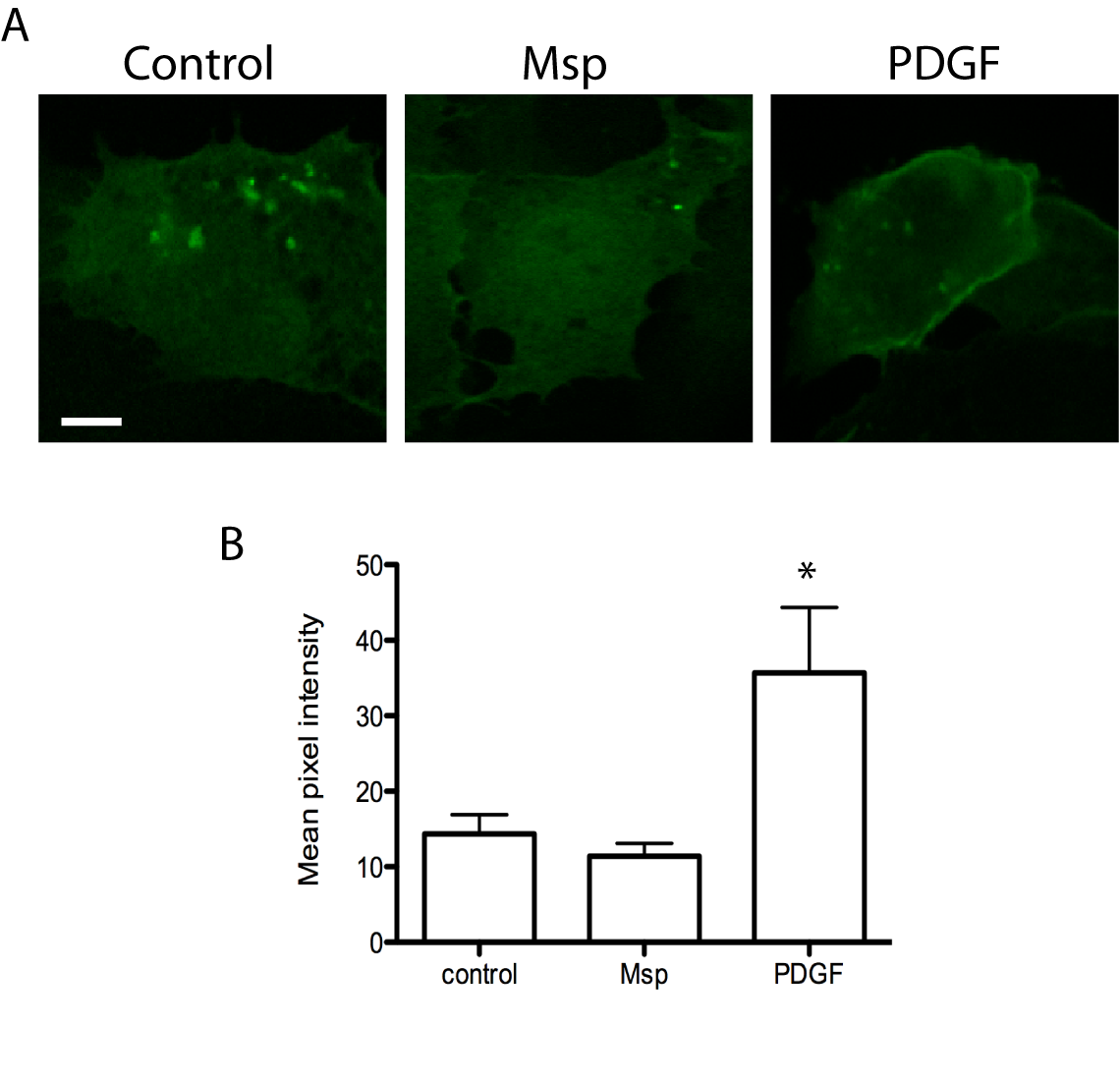

Supplement: Figure S1 — Localization of Akt-PH-GFP following Msp treatment. Rat-2 fibroblasts were transfected with Akt-PH-GFP prior to Msp treatment and fixation. Representative images are shown in panel A. (B) Fluorescence intensity at the plasma membrane was quantified using ImageJ. PDGF treatment serves as a positive control for recruitment to the plasma membrane. Graph represents mean ± SEM of 3 experiments (P<0.05). (TIF) [file pone.0023736.s001.tif]

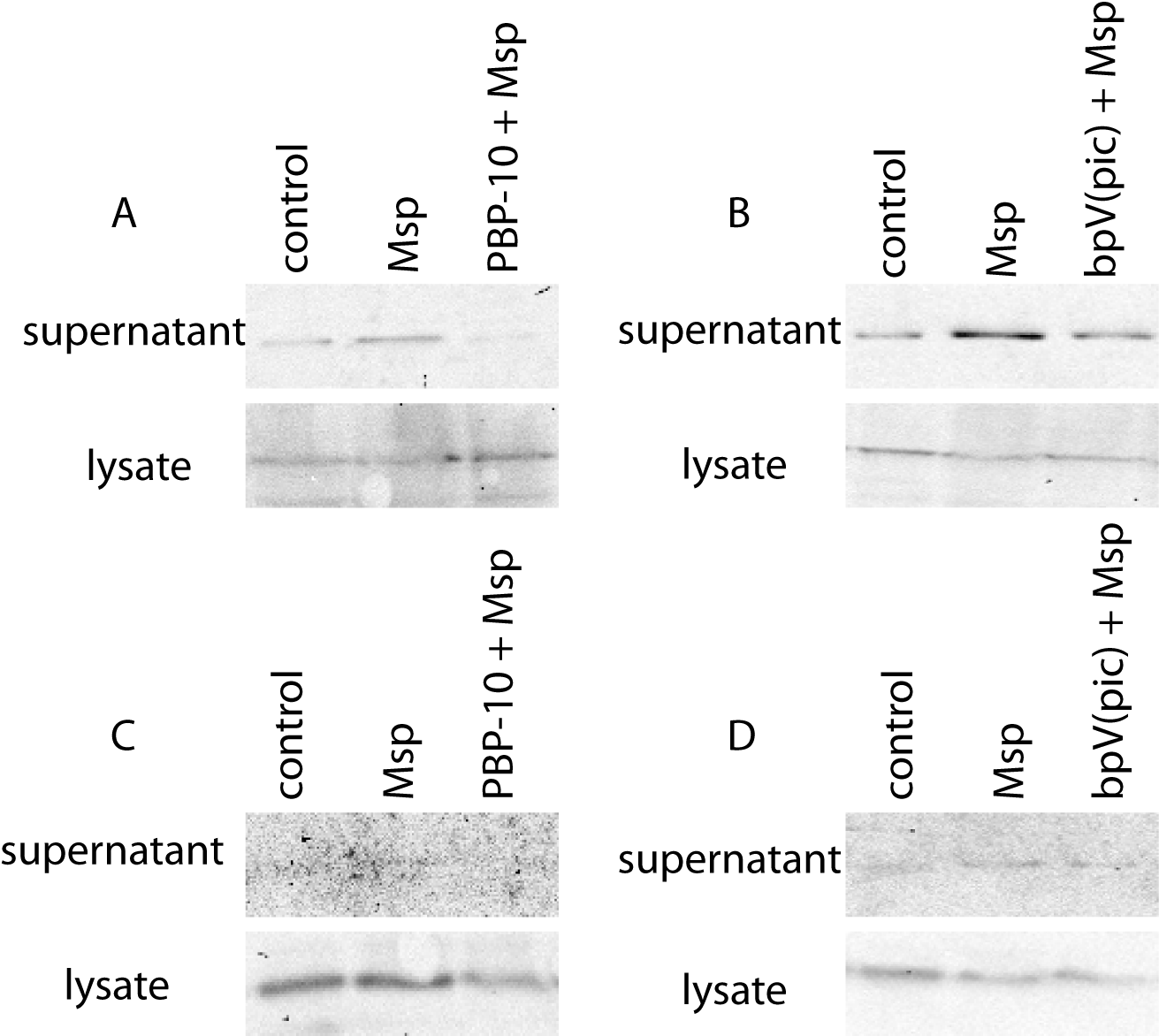

Supplement: Figure S2 — Treatment of fibroblasts with a PIP2 binding peptide or a lipid phosphatase inhibitor prevents Msp- mediated actin uncapping. Rat-2 cells were pretreated with (A, C) PBP-10 or (B, D) bpV(pic) prior to Msp treatment. Representative immunoblots of the amount of released gelsolin (A, B) and CapZ (C,D) are shown. (TIF) [file pone.0023736.s002.tif]
